# Structural Optimization of Microfluidic Chips for Enhancing Droplet Manipulation and Observation via Electrodynamics Simulation

**DOI:** 10.34133/cbsystems.0217

**Published:** 2025-03-06

**Authors:** Yanfeng Zhao, Zhiqiang Zheng, Jiaxin Liu, Xinyi Dong, Haotian Yang, Anping Wu, Qing Shi, Huaping Wang

**Affiliations:** ^1^ Intelligent Robotics Institute, School of Mechatronical Engineering, Beijing Institute of Technology, Beijing 100081, China.; ^2^ Department of Biomedical Engineering, City University of Hong Kong, Hong Kong 999077, China.; ^3^ Beijing Advanced Innovation Center for Intelligent Robots and Systems, Beijing Institute of Technology, Beijing 100081, China.; ^4^ Key Laboratory of Biomimetic Robots and Systems (Beijing Institute of Technology), Ministry of Education, Beijing 100081, China.

## Abstract

Digital microfluidic chips (DMCs) have shown huge potential for biochemical analysis applications due to their excellent droplet manipulation capabilities. The driving force is a critical factor for characterizing and optimizing the performance of droplet manipulation. Conducting numerical analysis of the driving force is essential for DMC design, as it helps optimize the structural parameters. Despite advances in numerical analysis, evaluating driving forces in partially filled electrodes remains challenging. Here, we propose a versatile electrodynamics simulation model designed to analyze the driving forces of partially filled electrodes to optimize the structural parameters of DMCs. This model utilizes finite element analysis to determine the voltage distribution within the DMC and calculates the driving force acting on the droplets using the principles of virtual work. Using this electrodynamics simulation model, we evaluated the effects of various structural parameters, including the dielectric constant and thickness of the dielectric layer, the dielectric constant and conductivity of the droplet, and substrate spacing, on the droplet driving force. This evaluation helps to optimize the structural parameters and enhances the droplet manipulation of DMCs. Measurements of droplet acceleration demonstrated that the droplet acceleration on the partially filled electrode aligns with the simulated driving force trend, which verified the effectiveness of the proposed electrodynamics simulation model. We anticipate that the electrodynamics simulation model is capable of evaluating the driving force in partially filled electrodes within complex DMCs, offering unprecedented possibilities for future structural designs of DMCs.

## Introduction

Microfluidics, which involves the precise manipulation of fluidic droplets at the microscale, has attracted widespread attention due to its ability to integrate traditional laboratory equipment into a “lab on a chip” [1–4]. Microfluidic systems can be broadly categorized into 2 types: continuous microfluidics and digital microfluidics. Continuous microfluidics relies on a steady flow of liquid [5,6], while digital microfluidics manipulates discrete droplets. Digital microfluidics, characterized by a high degree of automation, easy integration, high scalability, and flexible operation [7], is widely applied in biochemical experiments [8–16], medical diagnostics [17–23], and optical displays [24–28]. As the complexity of application tasks grows, additional functional modules, such as detection and heating modules, are integrated into digital microfluidic chips (DMCs) to broaden their application scope [20,29–31]. However, incorporating these additional functional modules reduces the available electrode area for droplet actuation, which diminishes the driving force required for efficient droplet movement. To mitigate the challenges posed by reduced electrode areas, it is crucial to conduct a numerical analysis of the droplet driving force in partially filled electrodes. This analysis ensures that DMCs maintain optimal functionality throughout their design and fabrication.

Rapid advancements in computer technology have facilitated the development of various numerical analysis methodologies, enabling researchers to explore and optimize the performance of DMCs. These advanced methodologies have allowed researchers to investigate key parameters directly influencing DMC performance. For instance, Khanna et al. [32] studied how variations in dielectric layer material and thickness affect voltage distribution in DMCs. Building on this, Türk et al. [33] investigated how dielectric layer characteristics influence the minimum driving voltage required for droplet manipulation, combining numerical simulations with experimental validation. Similarly, Pyne et al. [34] analyzed the relationship between partially filled electrodes and the driving force on droplets. While these numerical simulations rely on the thermodynamic model to accurately predict voltage distribution and droplet shape changes, which calculate the driving forces of droplets based on surface tension, they do not account for the effect of the applied voltage frequency on the driving force. In practice, the applied voltage frequency has a marked impact on the droplet driving force. To address this limitation, researchers have turned to the electromechanical model, which treats DMCs as equivalent to capacitors and resistors. Researchers have established electromechanical models for DMCs and applied the virtual work principle to calculate driving forces. For example, Chen et al. [35] examined the influence of frequency and droplet conductivity on droplet actuation by establishing correlation equations between the threshold voltage and key DMC parameters. Similarly, Singh and Hawkins [36] utilized an electromechanical model to analyze the relationship between applied voltage and droplet velocity in an open digital microfluidic device. Although the electromechanical model helps analyze the effect of applied voltage frequency, it becomes less effective with partially filled electrodes. Therefore, developing a numerical analysis model that can effectively evaluate partially filled electrodes while accurately accounting for applied voltage frequency effects on the driving force remains a substantial challenge.

In this study, we employed a versatile electrodynamics simulation model to analyze the driving forces of partially filled electrodes, which combines finite element analysis with an electromechanical model. The electrodynamics simulation model involves 2 steps: first, simulating the voltage distribution of the partially filled electrode using a finite element analysis model and, second, calculating the driving force of the droplet on the partially filled electrode through an electromechanical model. This method not only precisely calculates the driving force of a droplet on partially filled electrodes but also reveals how the applied electric field’s frequency influences different driving forces. We systematically analyzed the impact of various key parameters on the driving performance of DMCs, including the dielectric constant and thickness of the dielectric layer, the dielectric constant and conductivity of the droplet, and substrate spacing. To validate the electrodynamics simulation model, the droplet acceleration was measured and compared with the simulation results, which confirmed the reliability of the electrodynamics simulation model. Our proposed electrodynamics simulation model effectively analyzes the droplet driving forces in partially filled electrodes, which helps to design complex DMCs and enriches the functionality of DMCs in various applications.

## Materials and Methods

**Fig. 1.**
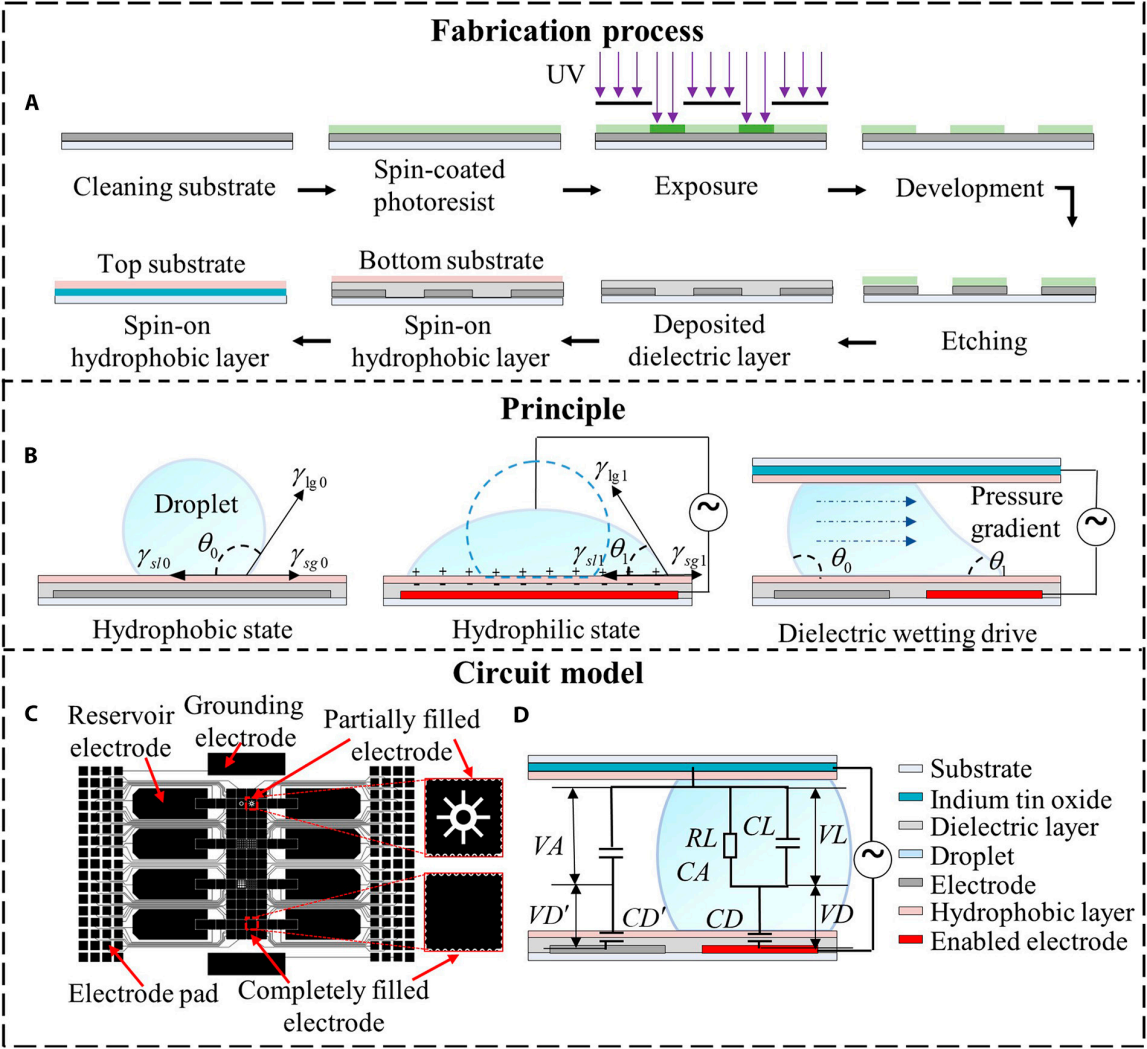
Schematic diagram of the fabrication process, working principle, and applications of a digital microfluidic chip. (A) Flowchart of the digital microfluidic chip substrate production process. (B) Schematic diagram of droplet actuation based on the principle of dielectric wetting (solid–liquid surface tension: $\gamma_{\mathrm{sl}0}$, $\gamma_{\mathrm{sl}1}$; liquid–gas surface tension: $\gamma_{\mathrm{lg}0}$, $\gamma_{\mathrm{lg}1}$; solid–gas surface tension:$\gamma_{sg0}$, $\gamma_{\mathrm{sg}1}$; contact angle: $\theta_{0}$, $\theta_{1}$). (C) Diagram illustrating the electrode distribution on a digital microfluidic chip. (D) Side-view schematic of the digital microfluidic chip with a circuit-model overlay. UV, ultraviolet.

As detailed in Fig. 1A, DMC fabrication begins with photolithography to create the bottom substrate, followed by dielectric material deposition. Both the top and bottom substrates are spin-coated with a hydrophobic layer. The DMC is then assembled by adhering the 2 substrates together with a spacer, forming a chamber where droplet manipulation is performed through dielectric wetting. Dielectric wetting refers to a phenomenon where an electrical potential applied between the electrode and the droplet alters the energy of the solid–liquid interface, thereby changing the contact angle of the droplet, as shown in Fig. 1B. Using dielectric wetting, the DMC adjusts the unilateral contact angle of the droplet, generating a pressure gradient that moves the droplet to the enabled electrode, as shown in Fig. 1B. A physical diagram of the DMC, with labeled electrodes for different functions, is shown in Fig. 1C. The DMC primarily consists of 4 components: substrate, electrode, dielectric layer, and hydrophobic layer, as shown in Fig. 1D. The dielectric layer plays a crucial role by isolating the electrodes from the droplets, preventing droplet electrolysis. Simultaneously, the hydrophobic layer enhances fluid flow and protects the DMC from contamination. Notably, partially filled electrodes change only the electric field distribution within the DMC without affecting the intrinsic electrical properties of the dielectric layer and the droplet. Therefore, we employed the electromechanical model to construct the equivalent circuit of the DMC, as shown in Fig. 1D. This model facilitates the calculation of the electrical parameters of both the dielectric layer and the droplet. Given the thinness of the hydrophobic layer and its minimal effect on the driving force of the DMC, it is excluded from the analysis in this section. The electrical parameters of the dielectric layer and the droplet are calculated as follows [37]:

$$\begin{array}{c} \mathrm{CD}=\frac{\varepsilon_{0}\varepsilon_{d}\mathrm{xy}}{d},\;CD^{\prime }=\frac{\varepsilon_{0}\varepsilon_{d}\left(L-x\right)y}{d},\\ \;\mathrm{CL}=\frac{\varepsilon_{0}\varepsilon_{l}\mathrm{xy}}{D},\;\mathrm{CA}=\frac{\varepsilon_{0}\varepsilon_{\mathrm{air}}\left(L-x\right)y}{D} \end{array}$$ (1)

$$\mathrm{RL}=\frac{\sigma_{l}\mathrm{xy}}{D}$$ (2)

where *CD* is the equivalent capacitance of the droplet-side dielectric layer, $CD^{\prime }$ is the equivalent capacitance of the air-side dielectric layer, *CL* is the equivalent capacitance of the droplet, *CA* is the equivalent capacitance of air, *RL* is the equivalent resistance of the droplet, $\varepsilon_{0}$ is the vacuum dielectric constant, $\varepsilon_{\mathrm{air}}$ is the dielectric constant of air, $\varepsilon_{l}$ is the dielectric constant of the droplet, $\varepsilon_{d}$ is the dielectric constant of the dielectric layer, *d* is the thickness of the dielectric layer, $\sigma_{l}$ is the conductivity of the droplet, *y* is the width of the electrode, *L* is the length of the electrode, *D* is the substrate spacing, and *x* is the droplet length along the direction of motion.

According to the principle of virtual work, the total energy storage *E* of a partially filled electrode is calculated as follows [37]:

$$E=\frac{1}{2}\left({\text{CDVD}}^{2}+{\text{CLVL}}^{2}+{\text{CAVA}}^{2}+CD^{\prime }V{D^{\prime }}^{2}\right)$$ (3)

where VD is the voltage of the dielectric layer on the droplet side, VL is the voltage of the droplet, VA is the voltage of the air, and $VD^{\prime }$ is the voltage of the dielectric layer on the air side.

The total driving force *F* acting on the droplet in the horizontal direction is expressed as follows [37]:

$$F=\frac{\partial E}{\partial x}=\frac{1}{2}\varepsilon_{0}y\left(\frac{\varepsilon_{d}}{d}{\mathrm{VD}}^{2}+\frac{\varepsilon_{l}}{D}{\mathrm{VL}}^{2}-\frac{\varepsilon_{\mathrm{air}}}{D}{\mathrm{VA}}^{2}-\frac{\varepsilon_{d}}{d}V{D^{\prime }}^{2}\right)$$ (4)

**Fig. 2.**
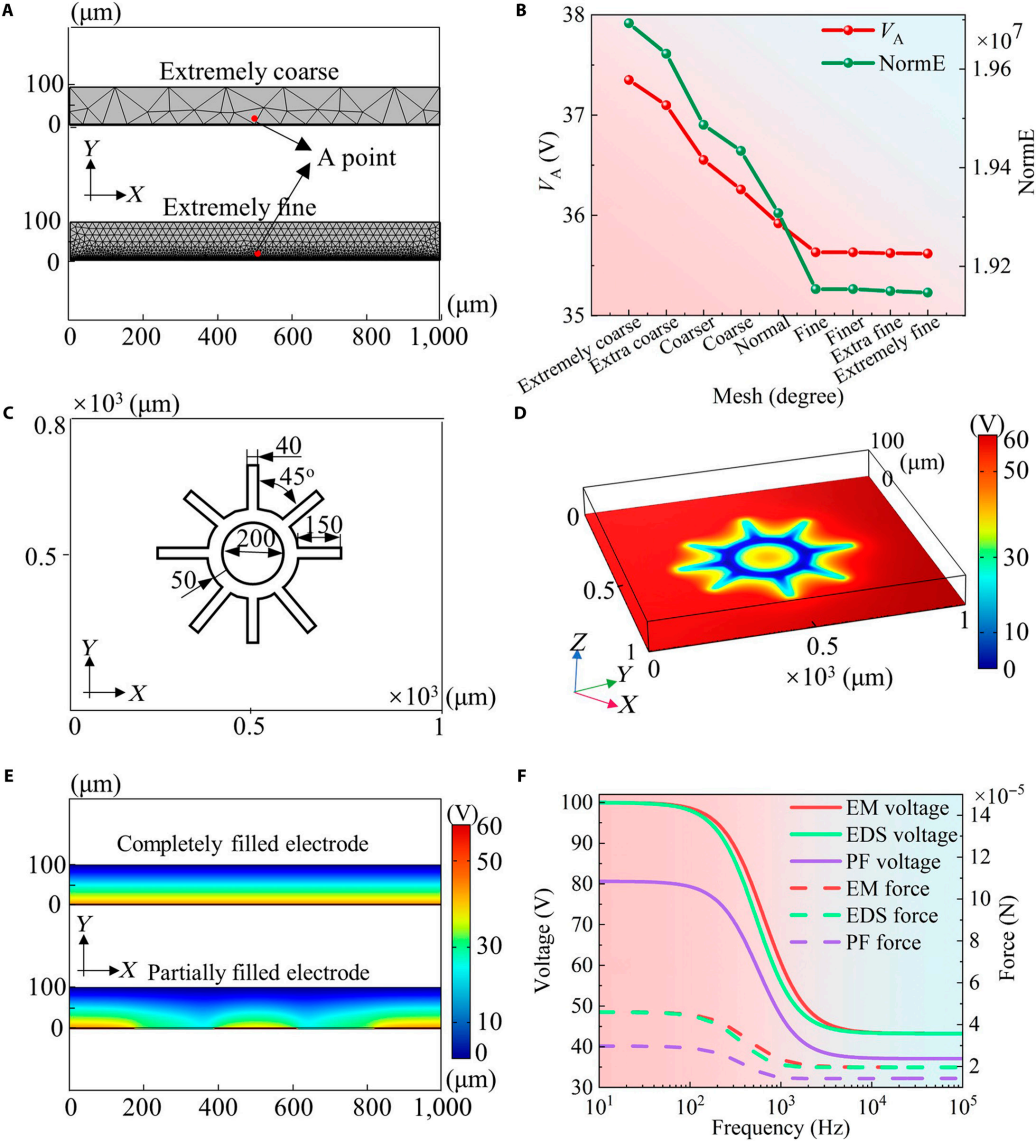
Numerical model and simulation results for the digital microfluidic chip. (A) Cross-sectional comparison of the extremely coarse and extremely fine mesh divisions in the electrical geometric model of a partially filled electrode. (B) Comparison of calculation accuracy at different mesh division levels. (C) Diagram illustrating the dimensional annotations of the missing pattern in the electrode. (D) Voltage distribution on the partially filled electrode. (To enhance the visibility of the potential distribution across the chip, the color bar range was adjusted to 0 to 60 V when exporting the results from COMSOL.) (E) Cross-sections of the simulated voltage distribution for completely and partially filled electrodes. (F) Comparison of voltage and droplet driving force values obtained from 2 analysis models (EM model: electromechanical model; EDS model: electrodynamics simulation model; PF electrode: partially filled electrode).

From the analysis, we derived a formula for the driving force acting on a droplet in the DMC. A key factor in this calculation is the voltage distribution within the DMC, which directly affects the driving force. To determine this voltage distribution, we developed a 3-dimensional electrical model of the partially filled electrode (Fig. S1a to c). The model, using an alternating current/direct current setting and specific simulation parameters (Table), allowed us to calculate the electric field distribution of the electrodes through finite element analysis. Simulation accuracy depends on the number of mesh elements, where increasing the number of mesh elements reduces simulation errors. However, this improvement comes at the cost of higher memory usage and longer computation times. To balance accuracy and computational efficiency, we optimized the mesh density in our 3-dimensional electrical model (Fig. 2A). At each mesh density, we calculated the electric field intensity at point A and the sum of the electric field intensity mode (normE) across the dielectric layer. The results, illustrated in Fig. 2B, indicate that both *V*_A_ and normE converge as the mesh becomes finer. This convergence allowed us to determine a mesh density that provided an optimal balance. We then simulated voltage distributions for both completely and partially filled electrodes using finite element analysis. The missing pattern of partially filled electrodes is shown in Fig. 2C, with the corresponding electric field distribution in Fig. 2D. To explore the effect of the electrode missing pattern on voltage distribution, we compared the cross-sections of completely and partially filled electrodes, as shown in Fig. 2E. The comparison reveals marked differences between the 2 voltage distributions. To ensure the reliability of our simulations, we compared the average voltage and droplet driving forces of completely and partially filled electrodes with values obtained from an electromechanical model. As depicted in Fig. 2F, the voltage and driving force curves for completely filled electrodes from the electrodynamics model closely align with those from the electromechanical model. The maximum voltage error for completely filled electrodes was 3.6069 V, with a maximum driving force error of 0.351 × 10^−5^ N, confirming the accuracy of the electrodynamics model in this case. However, for partially filled electrodes, marked discrepancies were observed. The maximum voltage difference was 19.326 V, and the maximum driving force difference reached 1.619 × 10^−5^ N. While the electromechanical model performs well for completely filled electrodes, it introduces large errors for partially filled electrodes. In contrast, the electrodynamics model accurately calculates voltage distributions for both completely and partially filled electrodes. By integrating finite element analysis with the electromechanical model, we overcome its limitations for partially filled electrodes, enabling more accurate droplet driving force calculations for electrodes at any missing level. Therefore, we adopted this combined approach to precisely calculate the driving forces for electrodes with arbitrarily missing levels.

**Table.** Parameters used in the numerical simulation of the digital microfluidic chip

Parameter	Value
$\varepsilon_{0}$	8.854 × 10^−12^ F/m
$\varepsilon_{\mathrm{air}}$	1
$\varepsilon_{l}$	80
$\varepsilon_{d}$	3.15
$\sigma_{l}$	5.5 × 10^−6^ S/m
*d*	3 × 10^−6^ m
*y*	1 × 10^−3^ m
*L*	1 × 10^−3^ m
*D*	1 × 10^−4^ m
*V*	100 V

## Results

Previous studies have demonstrated that several key parameters, the dielectric constant and thickness of the dielectric layer, the dielectric constant and conductivity of the droplet, and the substrate spacing of the DMC, affect droplet driving performance in DMCs [33,35,38]. However, the lack of simulation methods for partially filled electrodes has left the analysis of droplet driving performance in this context largely unexplored. To address this gap, we utilize a versatile electrodynamics simulation model to investigate how these parameters influence the droplet driving performance on a partially filled electrode in DMCs. While the droplet driving force fluctuates as the droplet moves across the electrode, it reaches its peak when the electrode is fully filled. For simplicity, we use the maximum droplet driving force as a reference point for comparison in the following discussion.

### The effect of the dielectric constant and thickness of the dielectric layer on the driving force

**Fig. 3.**
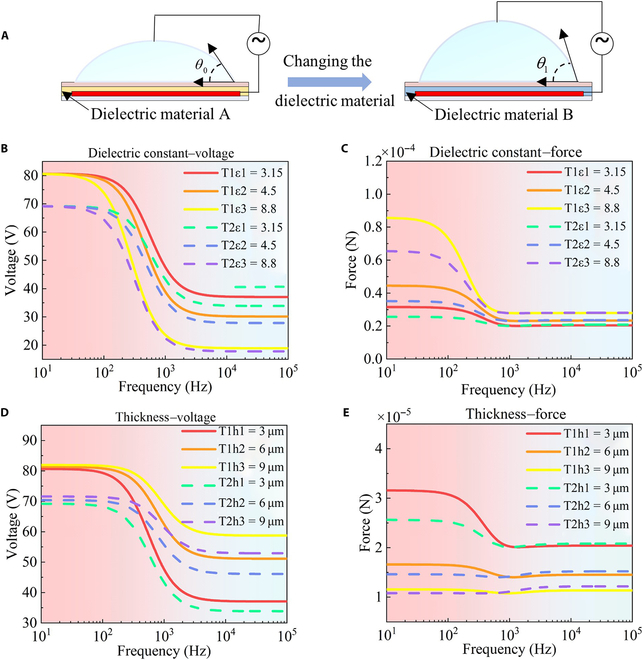
Investigation of the effect of dielectric constant and thickness of the dielectric layer on the droplet manipulation performance in a digital microfluidic chip. (A) Comparison of droplet contact angles under identical conditions, highlighting the influence of dielectric material on driving performance. (B) Comparison of voltages under varying dielectric constants for electrodes with distinct missing patterns. (C) Comparison of droplet driving forces under varying dielectric constants for electrodes with distinct missing patterns. (D) Comparison of voltages under varying thicknesses for electrodes with distinct missing patterns. (E) Comparison of droplet driving forces under varying thicknesses for electrodes with distinct missing patterns.

The dielectric layer is crucial for the functionality of DMCs due to its marked effect on their overall driving performance as shown in Fig. 3A. Two key parameters of the dielectric layer are particularly important: its dielectric constant and thickness. These parameters directly impact the performance of the dielectric layer, thereby affecting the driving force on the droplet. Therefore, it is essential to investigate how these parameters affect the driving performance. In this study, we used 3 widely used dielectric materials with varying dielectric constants: parylene C (3.15), silicon dioxide (4.5), and aluminum oxide (8.8). The thicknesses of the dielectric layers were set at 3, 6, and 9 μm, respectively. To examine the effect of the dielectric constant on the driving force, the thickness was maintained at 3 μm. Conversely, to investigate the effect of thickness on the driving force, the dielectric constant was kept constant at 3.15.

In our analysis, we examined the electric field distributions of 2 electrode types with distinct missing patterns: type 1, which has a radial shape, and type 2, which has a square array shape (Fig. S1b and c). These shapes were selected based on the bionic liver lobule vascular structure and biological array distribution to model arbitrary patterns. By calculating electric field distributions for these electrodes, we obtained voltage curves for dielectric layers with 3 different dielectric constants as shown in Fig. 3B. Notably, the voltage values for the 2 partially filled electrodes differed, but their voltage curves followed a similar trend with changes in frequency and dielectric constant. Specifically, the voltage of the dielectric layer decreased with increasing frequency, regardless of the dielectric constant. Similarly, at a constant frequency, the voltage of the dielectric layer decreased as the dielectric constant increased. Incorporating these voltage values into the driving force calculations, we obtained the driving force curve, as shown in Fig. 3C. These results show that for a given dielectric constant, the droplet driving force decreases with increasing frequency, indicating that low frequencies are beneficial for driving droplet motion. At 1,000 Hz, as the dielectric constant increased from 3.15 to 4.5 and then to 8.8, the driving force rose from 20.27 to 23.29 μN and finally to 27.92 μN. This demonstrates a positive correlation between the dielectric constant and droplet driving force. Therefore, regardless of the electrode type, low frequencies enhance droplet motion and a higher dielectric constant results in a stronger driving force.

By varying the thickness of the dielectric layer, the corresponding voltage curves, as shown in Fig. 3D, were derived. These curves show that the voltages decrease with increasing frequency across dielectric layers of different thicknesses. Notably, in the low-frequency band, changes in thickness have minimal impact on voltage. Additionally, at any frequency, a thicker dielectric layer results in a higher voltage. The droplet driving force, shown in Fig. 3E, indicates that for thinner dielectric layers, the droplet driving force decreases with increasing frequency. In contrast, for thicker dielectric layers (e.g., 6 and 9 μm), the capacitive effect slightly increases the driving force with frequency, although the overall driving force remains low. At 1,000 Hz, as the dielectric layer thickness of the first electrode increased from 3 to 6 μm and then to 9 μm, the corresponding droplet driving force decreased from 20.27 to 14.06 μN and finally to 10.78 μN. This demonstrates that the droplet driving force decreases with increasing thickness. Therefore, regardless of the partially filled electrode used, the droplet driving force is greater at low frequencies and with a thinner dielectric layer.

### The effect of the dielectric constant and conductivity of the droplet on the driving force

**Fig. 4.**
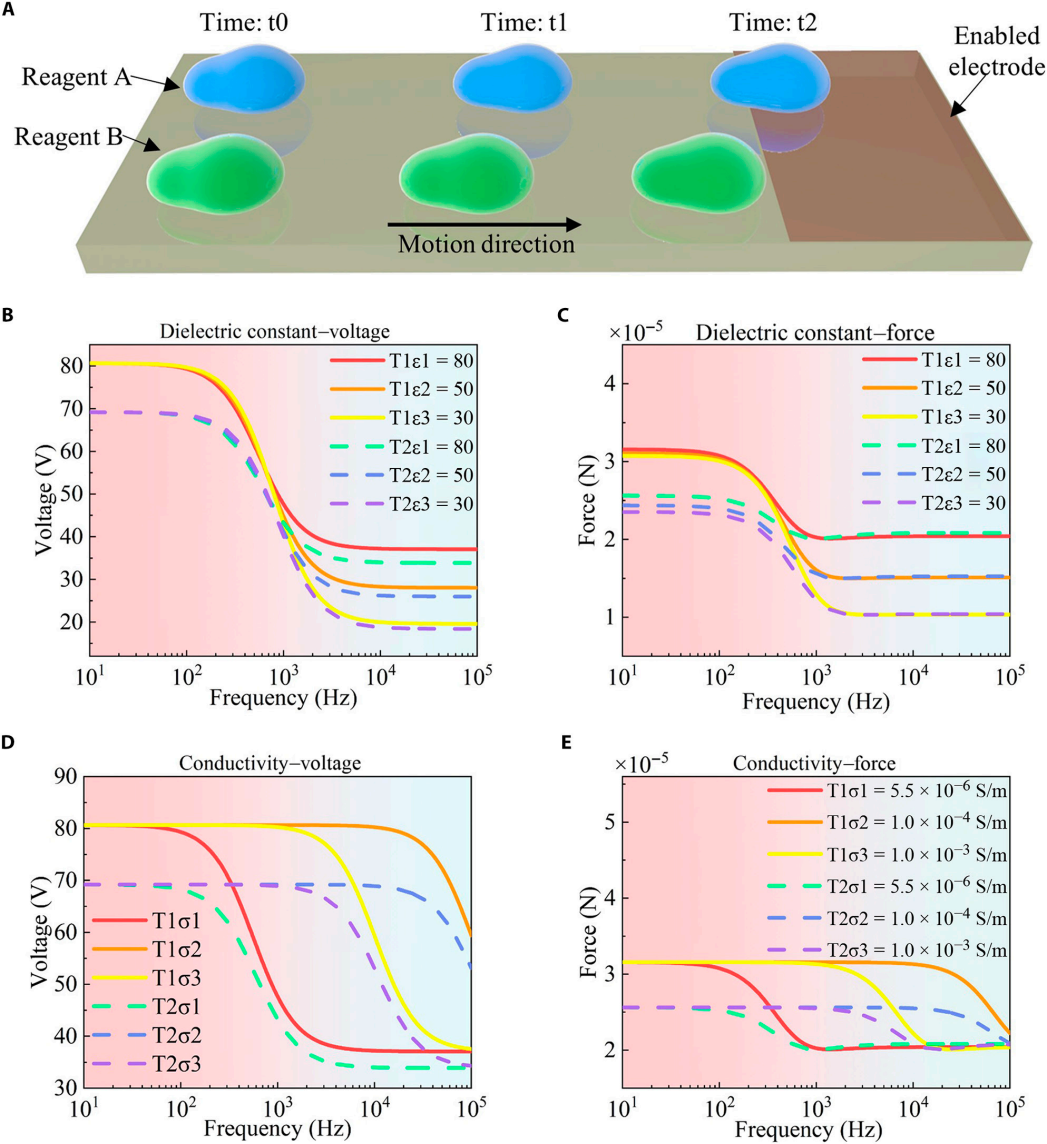
Investigation of the effect of the dielectric constant and conductivity of the droplet on the droplet manipulation performance in a digital microfluidic chip. (A) Displacement comparison of reagent droplets A and B under identical conditions, highlighting the influence of reagent properties on the performance of the digital microfluidic chip. (B) Comparison of voltages under varying dielectric constants for 2 electrodes with distinct missing patterns. (C) Comparison of droplet driving forces under varying dielectric constants for electrodes with distinct missing patterns. (D) Comparison of voltages under varying conductivity for electrodes with distinct missing patterns. (E) Comparison of droplet driving forces under varying conductivities for electrodes with distinct missing patterns.

In biochemical analysis using DMCs, effective manipulation of reagent droplets is critical for successful experiments. Notably, marked differences in the manipulation performance of various reagent droplets on DMCs, as shown in Fig. 4A, were observed (Movie S1). This section analyzes how the electrical properties of droplets, specifically the dielectric constant and conductivity, influence the driving force, offering insights into their performance on DMCs. For this analysis, we selected 3 reagent droplets with dielectric constants of 80, 50, and 30, respectively. The conductivity values were set to 5.5 × 10^−6^, 1 × 10^−4^, and 1 × 10^−3^ S/m, respectively. To examine the impact of the dielectric constant on the driving force, the conductivity value was fixed at 5.5 × 10^−6^ S/m. Similarly, to explore the impact of conductivity on the driving force, the dielectric constant was kept constant at 80.

By varying the dielectric constant of the droplet, the corresponding voltage curves, as shown in Fig. 4B, were derived. This figure shows that the voltage across the dielectric layer decreases with increasing frequency for droplets with different dielectric constants. In the low-frequency band, changes in the dielectric constant have minimal effect on the voltage. However, in the high-frequency band, a higher dielectric constant results in a substantially higher voltage. The corresponding droplet driving force, shown in Fig. 4C, demonstrates that the driving force decreases with increasing frequency under different dielectric constant conditions. This indicates that low frequencies are more beneficial for driving droplet motion. The figure shows that the dielectric constant has little effect on the driving force at low frequencies, but as the frequency increases, the driving force decreases more sharply for droplets with lower dielectric constants. When the frequency exceeds 10^4^ Hz, the droplet driving force stabilizes and remains constant. Therefore, regardless of the type of partially filled electrode used, the influence of the dielectric constant of the droplet on its driving force is negligible at low frequencies.

By varying the conductivity of the droplet, the corresponding dielectric layer voltage curves, as shown in Fig. 4D, were derived. The figure shows that in the low-frequency band, changes in frequency and conductivity have no discernible effect on the voltage. However, as the frequency increases, the dielectric layer voltage for droplets with low conductivity begins to decrease. The corresponding droplet driving force, shown in Fig. 4E, indicates that at low frequencies, the droplet driving forces with different conductivities are similar. As the frequency increases, the droplet driving force with lower conductivity decreases more rapidly. Therefore, for droplets with varying conductivities, maintaining the frequency before the turning point of the driving force ensures more efficient droplet movement.

### The effect of the substrate spacing on the driving force

**Fig. 5.**
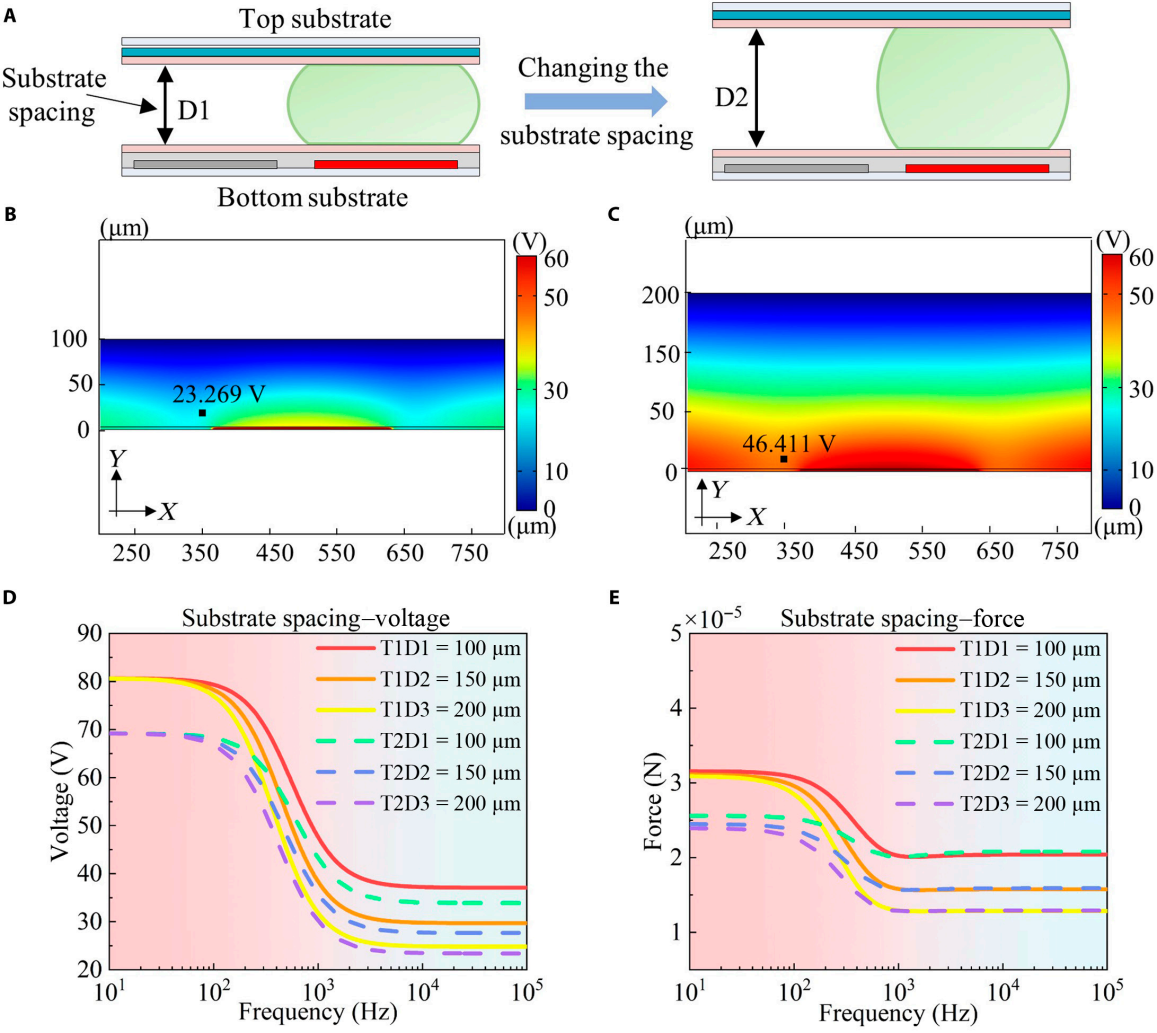
Investigation of the effect of the substrate spacing on the droplet manipulation performance in a digital microfluidic chip. (A) Schematic diagram of substrate spacing change of digital microfluidic chip. (B) Cross-sections of the simulated voltage distribution for partially filled electrodes at a substrate spacing of 100 μm. (C) Cross-sections of the simulated voltage distribution for partially filled electrodes at a substrate spacing of 200 μm. (D) Comparison of voltage under varying substrate spacing for electrodes with distinct missing patterns. (E) Comparison of droplet driving force under varying substrate spacing for electrodes with distinct missing patterns.

The spacing between the top and bottom substrates of DMCs, along with the electrode size, plays a dual role in determining both the volume and the driving force of the droplet, as shown in Fig. 5A. This section analyzes how changes in substrate spacing impact the driving force. For this purpose, we selected 3 widely used substrate spacings, 100, 150, and 200 μm, to establish a relationship between substrate spacing and voltage. The cross-sections of the electrode voltage distribution at varying substrate spacings are shown in Fig. 5B and C, illustrating how changes in substrate spacing alter the voltage distribution. We also analyzed the voltage distribution across different frequencies, as shown in Fig. 5D. This figure shows that the voltage decreases as frequency increases. At low frequencies, the voltage differences between various substrate spacings are minimal, while at high frequencies, reducing the substrate spacing leads to an increase in voltage. The relationship between substrate spacing and droplet driving force is shown in Fig. 5E, indicating that the trends for substrate spacing and driving force align with those for substrate spacing and voltage.

### Experimental verification of droplet drive performance

To validate the reliability of the simulation results, corresponding droplet driving experiments were conducted in DMCs. Measuring the driving force directly on a droplet is challenging due to the simultaneous actions of several forces, including the driving, drag, and viscous forces. Each of these forces interacts in complex ways that complicate isolated measurements. According to Newton’s second law, the acceleration of an object is directly proportional to the net external force applied to it. Because droplet acceleration is easier to measure, it serves as an effective indicator of the total driving force. As a result, droplet acceleration is used as an alternative metric to evaluate driving performance.

**Fig. 6.**
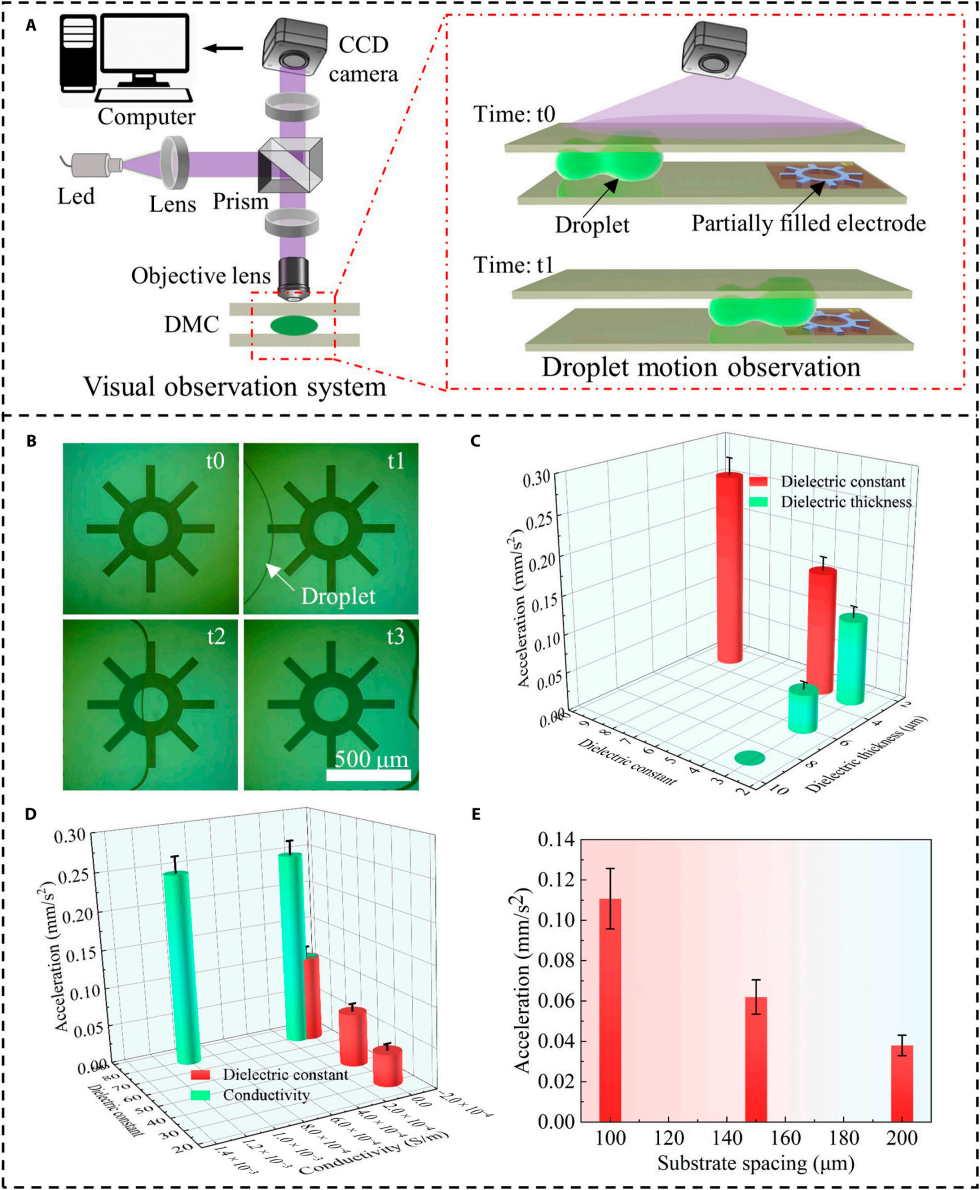
Experimental verification. (A) Diagram of the configuration of the visual observation system. (B) Motion process of a droplet in a digital microfluidic chip at different time points. (C) Effect of dielectric constant and thickness of the dielectric layer on droplet acceleration. (D) Effect of dielectric constant and conductivity of the droplet on droplet acceleration. (E) Effect of substrate spacing on droplet acceleration. CCD, charge-coupled device; DMC, digital microfluidic chip; LED, light-emitting diode.

**Fig. 7.**
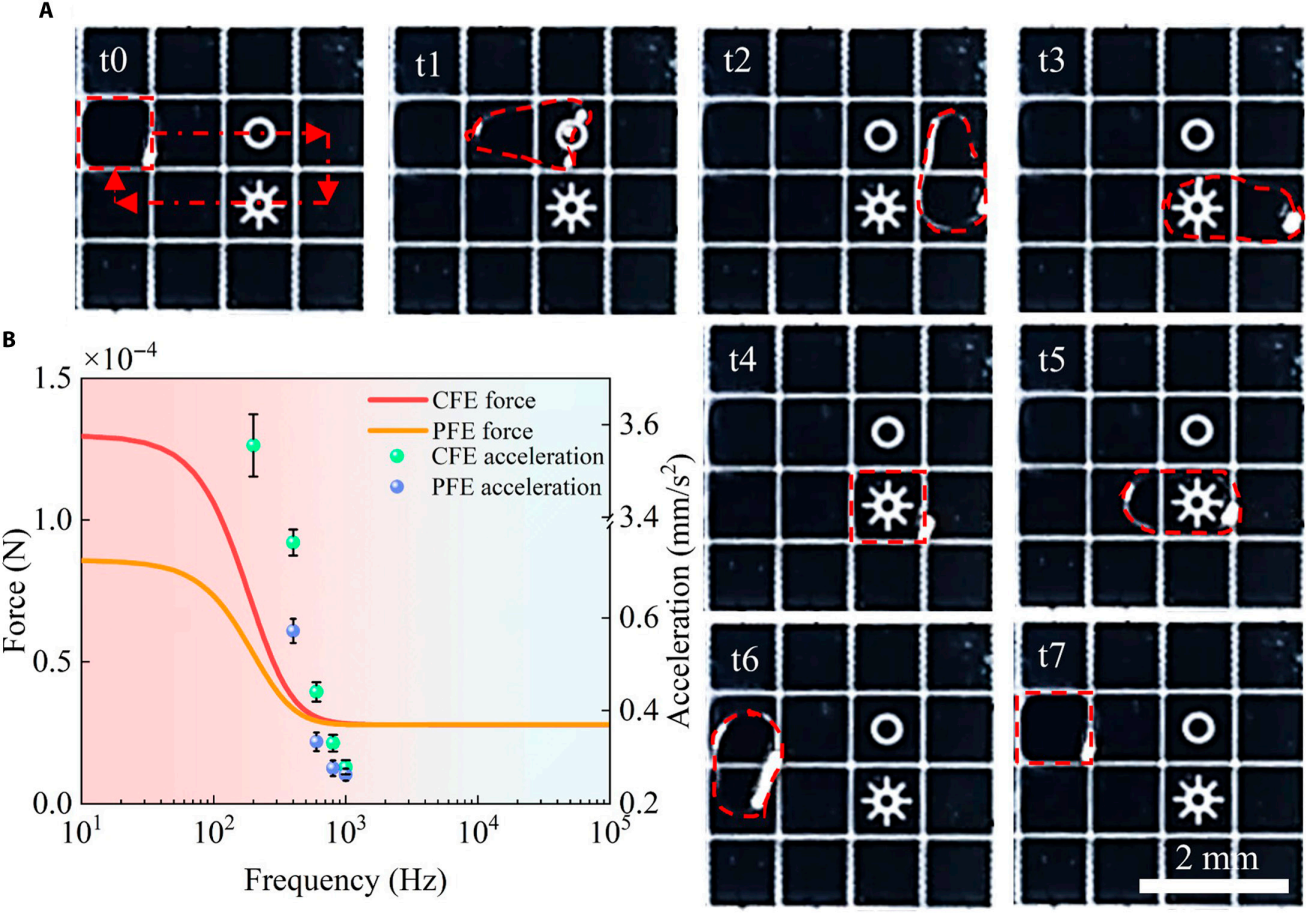
Droplet drive experiment. (A) Process of driving a droplet on completely and partially filled electrodes in a digital microfluidic chip. (B) Relationship curves between droplet acceleration and simulated droplet driving force at different frequencies (CFE force: completely filled electrode driving force; PFE force: partially filled electrode driving force; CFE acceleration: completely filled electrode driving acceleration; PFE acceleration: partially filled electrode driving acceleration).

A droplet motion observation system was constructed to capture droplet motion, as shown in Fig. 6A. The resultant droplet motion is shown in Fig. 6B. The droplet motion time was measured under various parameter conditions, and the acceleration of the droplet was calculated using Eq. (5). The average values of 10 repeated calculations are plotted in Fig. 6C to E. In Fig. 6C, the test results show that droplet acceleration decreases as the dielectric constant decreases or the dielectric layer thickness increases. Fig. 6D illustrates that a higher permittivity leads to a higher droplet acceleration, while increased conductivity initially raises acceleration, which then stabilizes. The droplet acceleration gradually decreases with increased substrate spacing as shown in Fig. 6E. These experimental results align with the simulation trends, thereby confirming the reliability of the simulation results. To further verify the universality of the simulation method, we used aluminum oxide with a thickness of 3 μm as the DMC dielectric layer. Droplet drive experiments were conducted with both completely and partially filled electrodes. The experimental results are depicted in Fig. 7A, showing the droplet moving along the dotted line. The droplet driving force curve and the corresponding experimental droplet acceleration data are shown in Fig. 7B. The absence of droplet acceleration indicates conditions where droplet motion is not driven. Both simulation and experimental results indicate that increasing the frequency decreases droplet driving force and acceleration. Under the same conditions, the simulated driving force and droplet acceleration of the completely filled electrode were greater than those of the partially filled electrode. The consistency between the experimental and simulation trends confirms the reliability of the simulation method and provides a valuable reference for future experimental research.

$$\bar{a}=\frac{2L}{t^{2}}$$ (5)

where $\bar{a}$ is the average acceleration of the droplet, *L* is the length of the electrode, and *t* is the droplet motion time.

### Biochemical application of partially filled electrodes

**Fig. 8.**
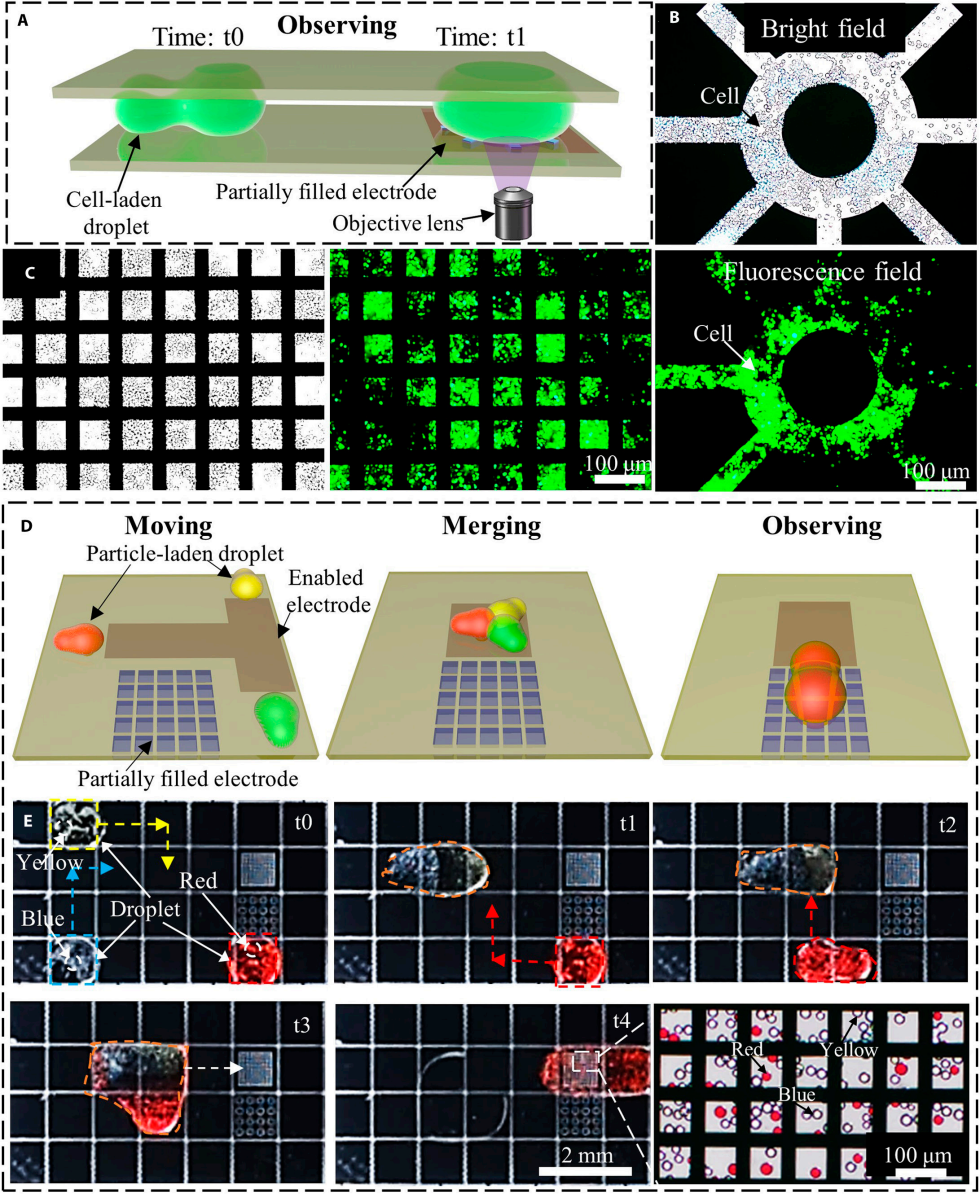
Application examples of partially filled electrodes in a digital microfluidic chip. (A) Application diagram of microscopic observation of partially filled electrodes in a digital microfluidic chip. (B and C) Observation images of cells on partially filled electrodes in a digital microfluidic chip under a bright field and a fluorescence field. (D) Schematic diagram of the movement, mixing, and observing of particle-laden droplets in a digital microfluidic chip. (E) Transport, merging, and microscopic observation of multiple-particle-laden droplets in a digital microfluidic chip (red particles, yellow particles, and blue particles).

Partially filled electrodes are versatile components that allow for the integration of various elements, such as detectors and heating modules, directly into the electrode structure. These electrodes also facilitate the imaging of droplet contents under a microscope, which is particularly advantageous in biochemical laboratories. Inverted microscopy is the standard platform for biochemical imaging. However, completely filled electrodes, often made from metals such as chromium or gold, are incompatible with inverted microscopy. As a result, indium tin oxide electrodes are used for such applications due to their transparency. Despite this advantage, indium tin oxide manufacturing is more complex and can present troubleshooting challenges. In contrast, partially filled metal electrodes are simpler to construct and compatible with inverted microscopy imaging, which makes them better suited for biochemical experiments. In this section, we demonstrate the use of partially filled metal electrodes in biochemical experiments. For instance, a schematic of a cell-laden droplet moving from a completely filled electrode to a partially filled electrode, observed via inverted microscopy, is shown in Fig. 8A. Cells on 2 different partially filled electrodes under bright and fluorescent fields are shown in Fig. 8B and C, demonstrating that cells are visible in both conditions. Additionally, a schematic of the movement, merging, and observation of droplets containing different particles on a DMC and the specific experiment is shown in Fig. 8D and E. Initially, particles of different colors were located in separate droplets. These droplets were moved and merged, and the mixed droplet was transferred to a partially filled electrode for observation (Movie S2). Enlarged images revealed particles in 3 different colors, illustrating the efficacy of partially filled electrodes in droplet manipulation for biochemical experiments and their compatibility with inverted microscopy. These examples highlight the advantages of using partially filled electrodes in biochemical experiments, showcasing their practicality and effectiveness in various applications.

## Discussion

In conclusion, we developed a versatile electrodynamics simulation model for DMCs to calculate the driving force of droplets with partially filled electrodes across different frequencies. By utilizing finite element analysis and an electromechanical model, we accurately determined the voltage distribution of partially filled electrodes and calculated the driving force of droplets. We conducted a comprehensive analysis of various parameters, such as the dielectric constant and thickness of the dielectric layer, the dielectric constant and conductivity of the droplet, and the substrate spacing of the DMCs, to understand their influence on the driving force. Our key findings indicate that lower-frequency bands are more effective at driving droplets. Specifically, in the low-frequency band, the driving force of the droplet is positively correlated with the dielectric constant of the dielectric layer and negatively correlated with its thickness. Additionally, the dielectric constant of the droplet has minimal effect on its driving force, and the conductivity of the droplet has little effect on the driving force until a turning point is reached. The greater the conductivity of the droplets, the greater the frequency at which the driving force transition occurs. The substrate spacing has a negligible influence on the droplet driving force in the low-frequency band. To validate our findings, we conducted droplet driving experiments, which confirmed the consistency between droplet acceleration and simulated driving force, thereby validating the reliability of our simulation outcomes. Furthermore, cell experiments using partially filled electrodes demonstrated that cells could be clearly observed under both bright and fluorescence field conditions. Overall, the numerical analysis method we have developed accurately predicts the driving force of droplets in DMCs. This method offers a valuable reference for DMC design and enhances the potential for further applications in biochemical experiments.

## Data Availability

All data supporting the findings in the paper and the Supplementary Materials are available from the corresponding authors upon reasonable request.
