# Supplementary material for: Structural Optimization of Microfluidic Chips for Enhancing Droplet Manipulation and Observation via Electrodynamics Simulation
Source: Cyborg Bionic Syst. 2025 Mar 6;6:0217. doi: 10.34133/cbsystems.0217 (PMC11884587; doi:10.34133/cbsystems.0217)
Supplement: Supplementary 1 — Figs. S1 and S2 Movies S1 and S2 [file cbsystems.0217.f1.zip › supplymentary material.docx]

Structural Optimization of Microfluidic Chips for Enhancing Droplet Manipulation and Observation via Electrodynamics Simulation

Yanfeng Zhao,^1^ Zhiqiang Zheng,^2^* Jiaxin Liu,^1^ Xinyi Dong,^1^ Haotian Yang,^1^ Anping Wu,^1^ Qing Shi^3^, Huaping Wang^4*^

Supplementary Materials

Affiliations

1. Intelligent Robotics Institute, School of Mechatronical Engineering, Beijing Institute of Technology, Beijing 100081, China.
2. Department of Biomedical Engineering,

City University of Hong Kong, HongKong 999077, China

1. Beijing Advanced Innovation Center for Intelligent Robots and Systems, Beijing Institute of Technology, Beijing 100081, China.
2. Key Laboratory of Biomimetic Robots and Systems (Beijing Institute of Technology), Ministry of Education, Beijing 100081, China.

* Corresponding author. Email: zhzheng@cityu.edu.hk, wanghuaping@bit.edu.cn

**1. Three-dimensional electrical simulation model of partially filled electrodes in digital microfluidic chip**

Three-dimensional electrical model of the completely and partially filled electrode were generated in COMSOL Multiphysics, as shown in Fig. S1. The model length (X-axis), width (Y-axis), and height (Z-axis) are set to 1000 μm, 1000 μm, and 100 μm, respectively. The electrode voltage is applied to the bottom substrate electrode, while the top substrate electrode is grounded. The missing electrode portion is electrically insulated. All other boundaries, except for the electrodes, including the boundary conditions of the droplets, are electrically insulated. The potential distribution on the electrodes was obtained through simulation. The voltage across the dielectric layer was calculated by subtracting the average potential of the dielectric layer from the applied voltage. The droplet voltage was then determined by subtracting the ground potential from the average potential of the dielectric layer. The alternating current/direct current (AC/DC) module was used to model the electric field, as it satisfies the governing equations assumed:

$\nabla\cdot J=Q_{j,v}$ (1)

$J=\sigma E+j\omega D+J_{e}$ (2)

$E=-\nabla V$ (3)

Where $J$ is the current density, $Q_{j,v}$ is the source term of the current density, $\sigma$ is the conductivity of the dielectric layer material, $E$ is the electric field strength, $\omega$ is the angular frequency, $D$ is the potential shift vector, $J_{e}$ is an applied current density source, $V$ is the potential.


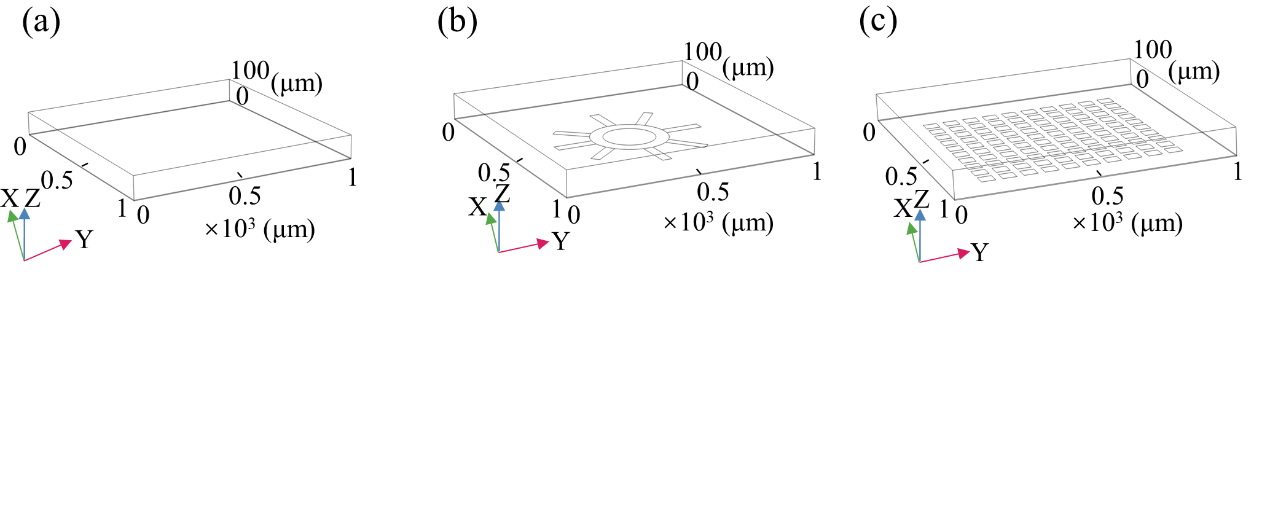


**Fig. S1 (a) Three-dimensional electrical model of the completely filled electrode.** **(b)** **Three-dimensional electrical model of a radial shape partially filled electrode of type 1.** **(c)** **Three-dimensional electrical model of a square array shape partially filled electrode of type 2.**

**2. The dimensional annotations and voltage distribution of type 2 the partially filled electrode**

**
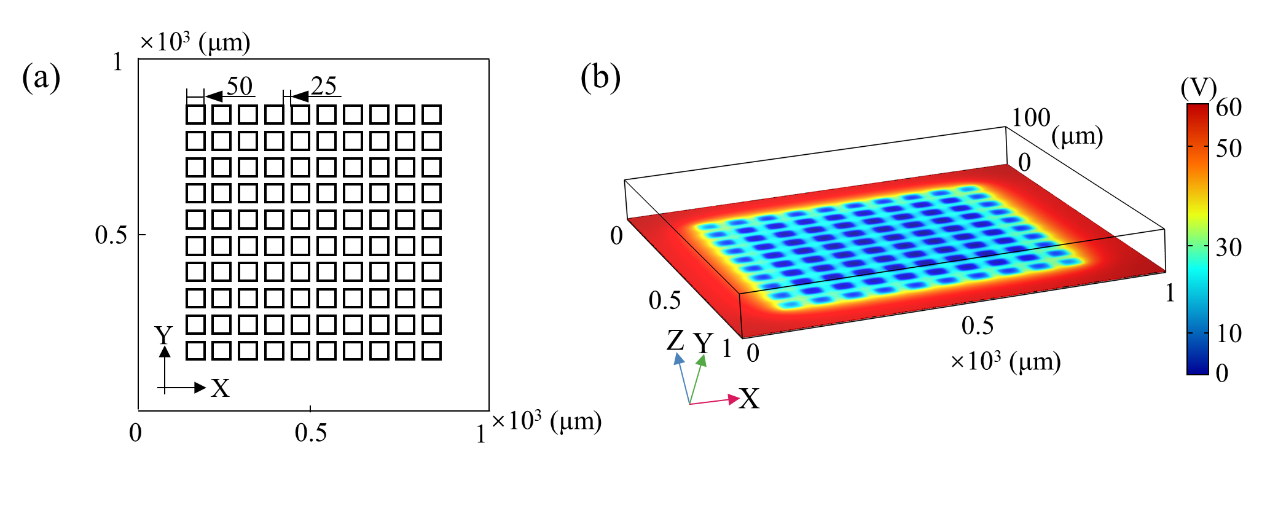
**

**Fig. S2 (a) Diagram illustrates the dimensional annotations of the missing pattern in the electrode. (b) The voltage distribution on the partially filled electrode.**
